# Report on the Means of Improving the Sanitary Condition of Chicago

**Published:** 1865-12

**Authors:** 


CHICAGO MEDICAL EXAMINER.

N. S. DAVIS, M.D., Editor.

VOL. VI. DECEMBER, 1865. NO. 12.

### Original Contributions.

ARTICLE XXXIX.

### REPORT ON THE MEANS OF IMPROVING THE SANITARY CONDITION OF CHICAGO.

To the Honorable Mayor and Common Council of Chicago:—The undersigned committee, appointed by a public meeting of the medical profession of this city, to recommend such measures as might be deemed important in relation to the sanitary condition of Chicago, more especially with reference to the anticipated prevalence of epidemic cholera, would, most respectfully, submit the following suggestions:—

That there are good and sufficient reasons for apprehending an epidemic prevalence of cholera in this country during the coming spring and summer, few who are conversant with the history of former epidemics of that disease, will be disposed to doubt. Hence, it is not only the part of wisdom and ordinary prudence, but it is the imperative duty of all municipal authorities to institute immediate and well-directed measures for preventing its prevalence, or mitigating its severity. While we freely admit that the primary or efficient cause of cholera may be some obscure and hitherto uncontrolled condition of the atmosphere, yet we claim that no fact in human history is better established than that local conditions exert a controlling influence in determining both the number of attacks and their fatality.

The local conditions known to be most efficient in promoting the prevalence and fatality of the disease may be included under the following heads, namely, *impure* and *damp air; unwholesome water; unwholesome food*, whether animal or vegetable; and excesses in the use of stimulating drinks, with neglect of personal cleanliness. That all these conditions exist in this city at the present time, in a highly aggravated degree, no reasonable man will deny. The naturally level surface of the soil, with an understratum of impervious clay, coupled with the very great neglect of occupants and owners of lots to connect them properly with the street sewers, causes the constant retention of sufficient surface water to impregnate the air with an excess of aqueous vapor; while the amount of animal and vegetable matter constantly left in the streets, alleys, gutters, and rear portions of lots, furnishes an abundance of noxious effluvia or gases to rise with the aqueous vapor, and, thereby, cause our city atmosphere to be both *impure* and *damp*. This is not only true of the *out-of-door* atmosphere, but is equally true of that in a large portion of the dwellings. Inspection will show that many hundreds of the houses in this city stand so directly over either standing water, or a soil so saturated, during all the spring, summer, and autumn months, that all the lower rooms are sufficiently damp to cause the rapid growth of fungi or mildew on books, clothing, and whatever is not used almost daily. This dampness, with its accompanying impurities, in our dwellings is a far more prolific source of disease, and especially of cholera, than the water and filth, so offensive to the eye and nose, in the open gutters of our streets. Hence, we shall earnestly call your attention to the measures necessary for remedying this evil.

Besides the accumulations of decomposable animal and vegetable matters, with water, in the streets, alleys, and surfaces of lots, the air of the city is further rendered impure by offensive emanations from sewers, cesspools, privies, stables, slaughtering, packing, and manufacturing establishments, &c., all of which are capable of being greatly mitigated, or entirely prevented, by the adoption of proper measures. The impure and unwholesome quality of much of the water used in this city is too well-known to need comment. That unwholesome food is often exposed for sale in our markets, and used by some classes of our citizens, is well-known; and no fact in the range of social science is better established, than that excessive use of alcoholic stimulants and personal uncleanliness very strongly predispose to attacks of cholera. Another very strong predisposing influence is found in the overcrowding of the sleeping apartments of boarding-houses and dwelling-houses of the laboring classes. Members of this committee can point to scores of small tenements in the central parts of our city, in the low and unventilated chambers (or more properly garrets) of which there are nightly just as many occupied beds as can be placed side by side, and leave room for a person to stand up between them. In this way, from twelve to fifteen adults are often confined in a quantity of air no more than sufficient to supply, properly, two. No intelligent physician, who practiced in this city during the cholera epidemics from 1849 to 1854, failed to observe the extremely injurious influence of such overcrowding. It was one of the most active influences in destroying thousands of the emigrants who crowded our city during those years. Having thus simply enumerated the chief conditions, now existing in our city, directly calculated to impair its sanitary condion at all times, and especially calculated to awaken any latent tendency to an epidemic of cholera, as well as to add ten fold to its malignity and fatality, when once begun, this committee will most respectfully and earnestly ask your attention to the following specific remedial measures:—

1st. The passage and rigid enforcement of such ordinances as will cause all lots, whether occupied or not, to be so connected with the street sewers and gutters as to relieve them from surface water, especially from under and immediately around dwellings. This is a matter of urgent importance in every section of the city, and as it can be properly done at the expense of the owners of lots, there is no reason why attention to it should be delayed.

2d. The immediate organization of a complete scavenger syssem, for the prompt removal of all garbage and refuse matter from every part of the city, and the vigilant enforcement of its practical operation. This, and this only, would prevent the further accumulation of filth and decomposable materials, and allow a reasonable time for the removal of what already exists. If every part of the city should be thoroughly cleaned and placed in good sanitary condition to-morrow, without the daily operations of a complete and efficient scavenger system, before the expected cholera season, next spring, every street and alley, with many of the lots, would be again reeking with filth and pestilential emanations. Hence, the very first money placed at the disposal of those having in charge the sanitary interests of the city should be applied to the establishment of a permanent and efficient scavenger system for the whole city.

3d. A more vigilant patrol of the river and its branches, for the purpose of excluding therefrom every species of animal and vegetable matter of a decomposable nature, except that which necessarily escapes from the street sewers.

4th. The more extensive inspection of privies, stables, street and lot gutters, the premises of slaughtering, rendering, packing, soap and candle, and all other manufacturing establishments, and the most rigid enforcement of cleanliness; together with the adoption of measures for intercepting and neutralizing of noxious gases from cesspools and sewers. For the latter purpose, charcoal placed in proper gratings over the openings and outlets of cesspools and sewers, affords an effectual and cheap material, as has been thoroughly demonstrated in some of the worst districts of London. For open gutters and privies, lime and other disinfectants should be used.

5th. The removal, as fast as possible, of all present accumulations of decomposable and offensive materials from the streets, alleys, and lots of the city. This task should be commenced and prosecuted with sufficient vigor that, with the daily operations of a scavenger system, the first warm spring days in April next should find all parts of the city clean and free from those materials that have in all ages and countries given to pestilence its virulence and fatality.

6th. The prevention of overcrowding and want of ventilation and cleanliness in boarding and tenant houses. This, in the present crowded state of our city, may not be practicable to the full extent, yet a proper system of inspection and judicious instruction to property owners, as well as occupants, would do much to mitigate the evil. There is one branch of this subject, however, which, in view of a probable epidemic of cholera, is of very great importance, and is fully under the legitimate control of the city authorities. All the members of this committee who practiced medicine here through all the cholera epidemics from 1849 to 1854, vividly remember how large a share of the entire mortality was occasioned by the tide of emigrants and new-comers, who, by every boat and railroad train, came to fill the small, damp, and filthy boarding-houses and dwellings; themselves not only unused to the climate, but often loaded with personal filth and filthy baggage. We speak advisedly, when we say that more than half of all the deaths occasioned by the epidemics of those years, occurred among this class of our population and those in immediate contact with them. To prevent a similar state of things the coming season, we would earnestly call your attention to the propriety of establishing adequate and comfortable sheds or temporary buildings at convenient places on all the Eastern and Southern lines of communication, entirely beyond the city limits, where, during all the cholera season, (if such season comes,) all emigrants, with their baggage, shall be detained long enough to have themselves and clothing washed and ventilated, and such as are sick among them properly cared for. In connection with this, such arrangements should be made with railroad managers, that persons of this class, whose destination was in the interior of the country, should be transported from the established places of detention directly through the city, without unnecessary delay. Such an arrangement could be made to serve all the useful purposes of a quarantine, and greatly lessen one of the most active causes of epidemic fatality in the city.

7th. The establishment of *Public Urinals*, at convenient distances, in all the more densely populated parts of the city, and the prohibition of the use of alleys and street corners for that purpose. The latter is not only an offence against public decency, but the impregnation of the soil in the alleys and around the street corners with urine is a prolific source of offensive and poisonous emanations. No species of animal matter more readily enters into decomposition, or emits elements more poisonous to the human system than urine. In connection with this, we would also strongly recommend the establishment of neat public fountains or drinking places. These would not only be a convenience to the poor and laboring classes, but they would deter thousands from resorting to the use of injurious and expensive liquids in the drinking saloons of the city.

8th. Finally, we would earnestly ask your immediate attention to the rigid exaction of certificates setting forth the cause of death, made by the attending physician, and returned to the proper officer, before permission is granted to bury the body. Such certificates should state clearly the age, sex, cause of death, and residence, signed by the physician. And in case no physician had visited the patient, that fact, with the probable cause of death, should be certified to by the sexton, and returned as in the other cases. All these certificates should be returned to a competent physician, who should see that they were properly made, placed on file, and properly tabulated for publication every week. By such a registry of deaths, it would be in the power of the city authorities to ascertain promptly, at all times, the occurrence of any unusual mortality in the city, and to trace it directly to the exact localities and classes of people giving rise to it; a matter of vital importance in every populous city. The present registry of deaths, so far as the *causes of the deaths is concerned*, is a ridiculous and shameful farce.

For the purpose of aiding in the prompt adoption and the judicious practical enforcement of the foregoing measures, we would suggest the propriety of selecting a permanent advising committee, consisting of five thoroughly competent physicians, who would be not only ready to aid the proper authorities in carrying out judiciously the measures already suggested, but would also be maturing ready for adoption, if the emergency should come, proper plans and locations for temporary hospitals for those attacked with cholera, suitable dispensaries for the prompt aid of such as might be threatened with attacks, and such other matters relating to the means of preventing and mitigating disease as the circumstances might require. Such a committee, properly selected, while they would cheerfully render their advisory services gratuitously, would be able not only to ensure the adoption of more judicious measures, and their more efficient enforcement, but would also save to the city thousands of dollars, by causing the avoidance of such confused, useless, and sometimes positively hurtful measures as are always liable to be adopted when these things are left until the alarm and excitement of a fatal epidemic is upon us.

In presenting the foregoing topics for your consideration, we have avoided all allusion to mere theoretical questions as to the contagiousness or non-contagiousness of cholera, and have made such recommendations only as are universally conceded to be necessary by all intelligent members of our profession, both in Europe and America. While we would not utter one word calculated to create needless alarm, we feel it our imperative duty to warn you, as the legal guardians of our city's welfare, that every fact connected with former epidemics, as well as every indication afforded by the progress of the present one up to this time, renders it almost certain that this continent will be the theatre of cholera during the year 1866.

And no mere pecuniary considerations should deter those in authority from the most diligent and best directed efforts to remove all the causes that either invite its prevalence or increase its severity. Our citizens could better afford to raise a fund of an hundred thousand dollars, five times repeated, than to suffer such an epidemic as would arrest all business for six months, depreciate the sanitary credit of our city for years, and depreciate, correspondingly, the value of property, to say nothing of the loss of human life. That the adoption and faithful execution of the measures recommended in this report would so far improve the sanitary condition of this city that the expected visitation of cholera would be rendered mild and comparatively harmless, we confidently believe. On the other hand, if these measures are neglected, and in their place only partial and spasmodic efforts made to clean out a few prominent streets and noted alleys, we just as confidently believe that before another Fourth of July comes, the insignia of mourning for the victims of cholera will be seen on every block in our city. But even if no cholera epidemic were ever to visit us, the sanitary measures recommended in this report, except simply that referring to quarantine stations and sheds for emigrants, would be imperiously demanded for protection against the annually increasing prevalence of cholera infantum, dysentery, and typhoid and typhus fevers, as well as for the comfort of our citizens, and the sanitary reputation of our city.

We therefore close this communication with an earnest prayer, that your honorable body will act on these subjects with that promptness and wisdom which should ever characterize those having the supervision of almost all that nearly 200,000 people hold valuable and sacred. And we fully pledge to your support, the best efforts of ourselves and our professional brethren of this city. All of which is most respectfully submitted.

Committee, {

N. S. DAVIS,

J. W. FREER,

J. P. ROSS,

H. HITCHCOCK,

RALPH N. ISHAM,

B. McVICKAR.

